# Cost-effectiveness of community-based type 2 diabetes prevention and control in Indonesia: a health economics modelling study

**DOI:** 10.1136/bmjph-2024-002161

**Published:** 2025-10-07

**Authors:** Rachmadianti Sukma Hanifa, M Rifqi Rokhman, Manuela Fritz, Vitri Widyaningsih, Ratih Puspita Febrinasari, Jaap A R Koot, Maarten J Postma, Jurjen van der Schans

**Affiliations:** 1 Department of Health Sciences, University of Groningen, University Medical Center Groningen, Groningen, Groningen, Netherlands; 2 Faculty of Pharmacy, Universitas Gadjah Mada, Yogyakarta, Daerah Istimewa Yogyakarta, Indonesia; 3 School of Social Sciences and Technology, Technical University of Munich, Munchen, Bayern, Germany; 4 Faculty of Medicine, Universitas Sebelas Maret, Surakarta, Central Java, Indonesia; 5 Division of Pharmacology and Therapy, Department of Anatomy, Histology and Pharmacology, Faculty of Medicine, Universitas Airlangga, Surabaya, East Java, Indonesia; 6 Department of Economics, Econometrics and Finance, Faculty of Economics and Business, University of Groningen, Groningen, GR, Netherlands; 7 Center of Excellence for Pharmaceutical Care Innovation, Universitas Padjadjaran, Bandung, West Java, Indonesia

**Keywords:** Community Health Planning, Diabetes Mellitus, Mass Screening, economics, Primary Prevention

## Abstract

**Introduction:**

Indonesia has implemented community-based health intervention (CBHI) to prevent and control type 2 diabetes mellitus (T2D) for over a decade and is in the process of scaling it up to reach a wider population. This study aimed to assess the cost-effectiveness of two CBHI scenarios: community-level T2D screening with or without health education.

**Methods:**

A model-based health economic evaluation, combining a decision tree and Markov model, was used to estimate the lifetime costs and quality-adjusted life years gained and to calculate the incremental cost-effectiveness ratio (ICER) of the two CBHI scenarios compared with a no-intervention. Simulations were run on a hypothetical cohort of 1183 people aged 40 without a prior diabetes diagnosis, from the payer’s perspective. Propensity score matching was employed to assess the effectiveness of health education. Data on transition probabilities, utilities, and health state costs were derived from the literature, while CBHI’s programme costs came from interviews. Scenario analysis evaluated the impact of repeating interventions over 10 years and varying the levels of CBHI uptake, referral to primary healthcare (PHC) adherence and diabetes knowledge gain needed to achieve cost-effectiveness. Sensitivity analyses assessed input parameter uncertainty.

**Results:**

With the current uptake (10.7%) and PHC referral adherence (6.0%), all one-time CBHI scenarios were cost-effective (ICER International dollars (Int$) 3550.28, and Int$8597.46 for T2D screening with or without health education, respectively). Scenario analysis demonstrated that repeated interventions remained cost-effective, and improving referral adherence and health education effectiveness enhanced cost-effectiveness. Sensitivity analysis showed a 60–88% probability that all CBHI scenarios are cost-effective within the 1 gross domestic product per capita threshold, with results being sensitive to cardiovascular disease risk among undiagnosed patients with T2D.

**Conclusion:**

CBHI scenarios offering T2D screening, with or without health education, are cost-effective. Enhancing coordination with PHC to manage newly detected cases and improving the quality of health education further improve cost-effectiveness.

**Trial registration number:**

NCT05239572.

**WHAT IS ALREADY KNOWN ON THIS TOPIC**

**WHAT THIS STUDY ADDS**

- Our study evaluates two CBHI strategies to prevent and control T2D using real-world CBHI implementation data from 1030 SUNI-SEA (Scaling-Up NCD Interventions in Southeast Asia) project participants in four districts in Indonesia: (1) screening for blood glucose and T2D risk factors only, and (2) screening combined with health education.
- Using a hypothetical modelled cohort of 1183 people, we demonstrate that the two CBHI strategies are cost-effective at the current implementation levels and could be cost-saving if the health education effectiveness were improved.

**HOW THIS STUDY MIGHT AFFECT RESEARCH, PRACTICE OR POLICY**

- Providing community-level T2D screening, with or without health education, is cost-effective. However, before scaling up the intervention, policymakers should improve the quality of health education and strengthen the coordination between CBHI and primary healthcare to ensure that follow-up confirmatory testing and treatment are both accessible and available.
- Further research is needed to enhance the effectiveness of health education at the community level, particularly in terms of its ability to change diabetes-related risk behaviours and its potential spillover effects.

## Introduction

 The burden of non-communicable diseases (NCDs), particularly diabetes, remains a pressing challenge for both the health and economic sectors, especially in low- and middle-income countries (LMICs). Over the past four decades, the global number of people with diabetes surged from 108 million in 1980 to 463 million in 2019, with approximately 80% of the cases occurring in LMICs.^1 2^ The global cost of managing diabetes is estimated to increase from US$1.3 trillion in 2015 to over US$2.1 trillion in 2030, even if the Sustainable Development Goals target to reduce NCD-related mortality by one-third is achieved.^3^

In Indonesia, the total diabetes prevalence increased from 6.9% in 2013 to 8.5% in 2018, with over 90% of cases estimated to be type 2 diabetes mellitus (T2D) and occurring among people aged 40 years and older.^4^ In 2016, managing T2D and its complications accounted for approximately 10% of total healthcare spending under the national health insurance, equivalent to US$567 million out of US$5055 million.^5 6^ Without sufficient intervention, the prevalence is projected to reach 16.1% by 2045, but could be reduced to 9.2% when the coverage of T2D prevention and control programmes is expanded, and public efforts to control T2D risk factors are improved.^7^

Indonesia’s current guideline for managing and controlling T2D recommends starting T2D screening among high-risk individuals or those aged 40 years and older.^8^ In line with this, the Indonesian government has established a community-based health intervention (CBHI), formerly known as *Posbindu,* since 2013.^9^ As part of ongoing health reform, *Posbindu* has been integrated into the broader *Posyandu* network, which now addresses a wider range of health needs across different age groups.^9 10^ Specifically for T2D control, *Posyandu* provides blood glucose and NCD risk factors screening, health education and referral of probable or high-risk T2D cases to primary healthcare (PHC) for diagnosis and treatment. These services are delivered by community health workers under the supervision of PHC staff.^9^,^12^

Delivering T2D prevention and screening through CBHIs, such as *Posyandu,* is effective and cost-effective. A recent meta-analysis indicates that individuals participating in CBHIs have a 46% lower risk of developing T2D compared with those in the control group.^13^ Additionally, delivering T2D screening within communities has been deemed more cost-effective than screening at PHC facilities because it is more accessible to the communities, thus reducing indirect medical costs.^14 15^

Despite the promising evidence regarding CBHI for T2D control and prevention, awareness and control of T2D in Indonesia remain low. In 2021, after a decade of implementing *Posyandu*, an estimated 14.3 million people with diabetes (74% of the total diabetes cases) remained undiagnosed.^16^ This condition increases the risk of complications due to prolonged high glycaemic levels.^17^ Additionally, between 2013 and 2018, risk factors such as abdominal obesity significantly increased from 27% to 31%, while adequate physical activity decreased from 88% to 79% nationwide.^18^ These trends may suggest potential implementation challenges that could compromise the real-world value of CBHI.

As part of the current health system transformation initiative, the Indonesian government aims to revitalise the *Posyandu* network across the country, as only 203 out of 514 districts have implemented the *Posyandu* programme in more than 80% of their PHC facilities.^19 20^ Nevertheless, in the context of limited healthcare resources, especially for preventive measures, decision-makers need to assess the value of CBHI and revisit it as new evidence on its implementation and utilisation emerges.^21 22^ While several studies promote community-level T2D screening or lifestyle interventions as cost-effective, most originate from non-Indonesian contexts, limiting their applicability due to differences in health system structures.^14 23^ Although a prior cost-effectiveness analysis of community-level T2D screening has also shown to be cost-effective in Indonesia, the study assumes an 80% follow-up rate for confirmatory testing, which may overestimate the result, considering the persistently high proportion of undiagnosed cases in the country.^2 15^

Therefore, this study aims to estimate the cost-effectiveness of CBHI for the prevention and control of T2D in Indonesia using real-world implementation data from four districts. The study is part of the ‘Scaling-Up NCD Interventions in Southeast Asia’ (SUNI-SEA) project, a global collaborative research initiative that seeks to validate the (cost)-effectiveness of strategies to tackle NCDs in Southeast Asia.^24^ In this context, the study specifically addresses the following research question: what is the cost-effectiveness of community-based blood glucose and NCD risk factor screening, either alone or combined with health education, followed by referral of probable or high-risk T2D cases to PHC for diagnosis and treatment among people aged 40 years in Indonesia?

## Methodology

A hybrid model combining a decision tree and a Markov model was employed to estimate the lifetime costs and health benefits of CBHI components for the screening and control of T2D. The analysis compared two strategies: community-level blood glucose and NCD risk factors screening, either alone or in combination with health education, followed by referral to PHC for high-risk or probable T2D cases for treatment or diagnosis. These were evaluated against a null scenario (no community-level screening and health education), following WHO-CHOICE (Choosing Interventions that are Cost-Effective) recommendations to use a no-intervention comparator to support sector-wide priority setting.^21^ The decision tree captured the short-term output of each scenario, while the Markov model extrapolated these outputs to estimate its long-term impact.

The analysis adopted a payer perspective, incorporating both direct medical costs associated with different health states and programme costs to establish a CBHI. Indonesia’s healthcare system operates under a tiered referral model, progressing from primary to secondary and tertiary levels.^25^ While patients may choose to bypass this system and pay out of pocket to access higher-level care directly, diabetes care is fully covered under the national health insurance scheme (JKN) when patients follow the referral pathway.^26^ Given that CBHI programmes are typically government-funded and that JKN currently covers over 90% of the population, adopting the payer perspective is appropriate for informing health resource allocation decisions.^26 27^

The model was simulated among a hypothetical cohort of 1183 people aged 40 without a prior diagnosis of diabetes, using a lifetime horizon and a 1-year cycle. The size of the cohort was estimated from the Indonesian demographic data based on the proportion of the population aged 40 years and older, along with the average number of villages and population per province.^28^,^30^ The starting age was based on the Indonesian guideline, which recommends T2D screening for ≥40 years old.^8^ A lifetime horizon was selected to adequately capture the benefits of early detection and health education, which often emerge later in life. A 1-year cycle was deemed appropriate to model chronic conditions, especially when the model aims to reflect the entire lifespan of the cohort.^31^

Both effects and costs were discounted at a rate of 3%, as recommended by the Indonesian Health Technology Assessment and the WHO CHOICE guideline.^21 32^ Transition probabilities, utility values and treatment costs were derived from systematic literature searches conducted separately for each parameter using PubMed. Priority was given to studies conducted in the Indonesian settings. Where data were unavailable, we extended the search to include Southeast Asian and broader Asian populations, with a preference for studies most aligned with the model’s context. The CBHI programme cost was obtained through in-depth interviews with local stakeholders as part of the SUNI-SEA project. The rate of CBHI’s uptake, adherence to PHC referral recommendations and the effectiveness of health education were calculated from the SUNI-SEA baseline survey collected in 2021. Information about the SUNI-SEA project and its sampling method is further explained in online supplemental figure A1. Summary of the SUNI-SEA Project.

### Model description

At the start of the decision tree model, individuals were categorised as either having undiagnosed T2D or non-T2D, which was further categorised as pre-diabetes or normal glucose tolerance (NGT) with low, medium or high risk T2D. Individuals accessing CBHI would undergo a random capillary blood glucose (RCBG) test, along with measurements of body mass index, waist circumference and blood pressure to categorise their T2D risk profile, as shown in table 1.^33^ Participants with RCBG levels exceeding 200 mg/dL or those categorised as high risk were referred to PHC for further diagnosis and treatment. Those adhering to the PHC referral recommendation were assumed to receive confirmatory tests and treatment through the *Prolanis* programme, which is a chronic disease management initiative for managing uncomplicated hypertension and T2D cases at the PHC level.^34^ Non-adherent participants with undiagnosed T2D were assumed to remain undiagnosed and would receive treatment later at specialist care when their glycaemic levels became even higher. Similarly, non-adherent high-risk individuals would not receive any medication to manage their risk.

**Table 1:** NCDs risk classification performed by CBHI^33^

	Healthy (low risk/green)	At risk of NCDs (moderate risk/yellow)	NCDs (high risk/red)
BMI	18.5–22.9	23–24.9	≥25
Waist circumferences	Men: ≤90 cm Women: ≤80 cm		Men: > 90 cm Women: >80 cm
Blood pressure	<130/80	130–139/80–89	≥140/90
Blood glucose level	80–144	145–199	≥ 200
IEC recommendations	General NCDs IEC. Screening every 3 years at CBHI.	IEC based on the condition. Screening every year at CBHI.	Referral to PHC for diagnosis and treatment.

BMI, body mass index; CBHI, community-based health intervention; IEC, information, education and counselling; NCDs, non-communicable diseases; PHC, primary healthcare.

Individuals with NGT who were false positives were assumed to be referred back to the CBHI and were assigned to the NGT group according to their T2D risk classification. Lastly, in the reference scenario of no community-level screening or health education, it was assumed that undiagnosed individuals would stay undiagnosed and untreated until they eventually were diagnosed and treated by specialist care. This assumption was based on the likelihood that the longer a diabetes case remains untreated, the higher glycaemic levels could be, which may necessitate combination therapies that only a specialist doctor can prescribe.^8^

At the end of the decision tree, individuals were categorised into one of seven groups: pre-diabetes, NGT with low, moderate or high risk (treated or untreated), diagnosed or undiagnosed T2D. Additionally, individuals were stratified by their exposure to health education. The categorised individuals were then progressed to a Markov model, which projected the lifetime costs and quality-adjusted life years (QALYs) of the CBHI strategies. The model included eight health states, allowing individuals to remain in the same state, transition to another or die due to age-adjusted all-cause mortality risk (online supplemental figures B1,B2. Illustration of the Decision Tree and Markov Model).^35 36^ The simulation continued until all individuals reached the death state.

The health education effect was modelled based on the improvement in T2D knowledge (ie, general T2D knowledge, risk factors, symptoms and complications), which was assumed to increase lifestyle index (ie, no-smoking, no alcohol consumption, adequate physical activity and adequate fruit and vegetable consumption).^37 38^ We used the SUNI-SEA baseline data to estimate the association between the T2D knowledge and the lifestyle index. The regression coefficient was used to predict whether the observed knowledge improvement would lead to the adoption of at least one new behaviour, thereby contributing to the lifestyle index and ultimately linked to a reduced annual risk of pre-diabetes or T2D. The risk reduction value was obtained from a previous study, and we assumed a similar risk reduction for pre-diabetes and T2D incidence.^38^

Additionally, we assumed that undiagnosed T2D individuals had a higher risk of cardiovascular disease (CVD), in line with prior studies that show a higher complication rate and increased risk of CVD among individuals with undiagnosed diabetes.^39 40^ CVD was chosen to represent T2D-related complications due to its highest prevalence and associated cost among the overall T2D complications in Indonesia, and the availability of local longitudinal data.^5 41^

### Effectiveness of different CBHI strategies

The base case scenario of the two CBHI strategies, that is, blood glucose and NCD risk factors screening with or without health education scenarios, was modelled with a 10.7% uptake and a 6.0% adherence rate to the PHC referral recommendation. Adherence to the referral recommendation rate was calculated based on the number of *Posyandu* participants who went to the PHC after being referred by community health workers during the *Posyandu* activity.^42^ The effectiveness of T2D screening was based on the diagnostic performance of RCBG.^43^ To estimate the impact of health education on T2D knowledge, we used propensity score matching (PSM) on the SUNI-SEA baseline data, comparing adults (≥40 years) without a prior diabetes diagnosis who attended *Posyandu* in the past 12 months with non-attendees having similar observable characteristics (table 2). A detailed explanation of the logit specification is provided in Online supplemental table C1, while the balance assessment of the PSM can be found in online supplemental table C2 and figure C1. Propensity Score Matching.

**Table 2:** Effect of participating in CBHI on diabetes knowledge improvement

Variable	ATET (SE)	Control mean	Observation
CBHI participation	0.860 (0.250)	8.930	1018

ATET, average treatment effect on the treated; CBHI, community-based health intervention; Control, non-CBHI participants.

### CBHI and treatment cost

CBHI costs encompassed programme and intervention expenses. The programme costs included the average total expenditure to provide training and yearly incentives for five community health workers, which are necessary for establishing one CBHI. Intervention costs comprised expenses to develop health education materials and the procurement of RCBG kits per individual screened. Detailed programme cost to establish one CBHI can be seen in online supplemental table D1. Programme and Intervention Cost.

Treatment costs covered the direct medical expenses associated with each health state. Direct medical costs for treating diagnosed T2D at the secondary care level and CVD complications were derived from the National Health Insurance claim data for patients with T2D, as reported in a previous study.^5^ The direct medical cost for treatment at the PHC level was estimated by summing the yearly capitation fee within the PHC sector, the *Prolanis* programme cost per capita, the average cost of hospitalisation and the treatment cost as reported in previous studies.^5 34 44^ No direct medical costs were assigned to individuals with NGT, with pre-diabetes or with undiagnosed T2D. However, high-risk NGT individuals who adhered to the PHC referral recommendation were assumed to be enrolled in the *Prolanis* programme for diabetes risk management and would therefore incur the yearly *Prolanis* programme costs.^34^ All costs were presented in international dollars (Int$) for the year 2022 (1Int$=IDR4850.70).^45^

### Outcome measures

The primary outcome of the analysis was the incremental cost-effectiveness ratio (ICER), calculated as the difference in expected total costs divided by the difference in expected total QALYs gained from each CBHI strategy compared with the reference scenario. Both the expected total costs and QALYs were estimated using 10 000 Monte Carlo Simulations (MCS), in line with current academic recommendations that support using probabilistic analysis, rather than deterministic analysis, as the basis for the base case results.^46 47^

QALYs associated with each health state were assessed using the EQ-5D-5L instrument, applying the Indonesian-specific time trade-off value set, which was derived from the previous studies.^48^,^50^ The ICER was then compared with a willingness to pay threshold of 1 to 3 times Indonesia’s gross domestic product (GDP) per capita (1 GDP per capita in 2022=Int$14 445.7) to determine the cost-effectiveness of each CBHI strategy, in line with WHO CHOICE and the Indonesian Health Technology Assessment guidelines.^21 32 51^

### Scenario analysis

Three scenario analyses were conducted. First, individuals without T2D classified as pre-diabetes, moderate or high-risk NGT from the initial screening were rescreened annually, while those classified as low-risk NGT were rescreened every 3 years, instead of just once as in the base case scenario (table 1).^33^ Those who were false positives but confirmed to have pre-diabetes were also rescreened annually. Additionally, undiagnosed T2D individuals who had false negatives were assumed to enter the high-risk category and receive annual rescreening. Adherence to rescreening was assumed to be equal to the follow-up rate for diagnosis and treatment after screening, which was set at 6.0% across all groups and modelled only for the first 10 years of the simulation. Second, we examined how varying the CBHI’s uptake and adherence to PHC referral recommendation rates, from 5% to 100%, would impact the value of the ICER of all the CBHI strategies. Lastly, we explored the level of T2D knowledge improvement needed to enhance the lifestyle index, thereby reducing T2D risk and making the CBHI cost-saving.

The model was constructed using Microsoft Excel 365. To check internal validity, we verified the accuracy of the formulas in each cell. We also tested the model by setting the effects of health education and the uptake level of CBHI to zero. In this scenario, the total health gain and treatment cost were similar to those of the null scenario, confirming that the formulas were correct.

### Sensitivity analysis

Probabilistic sensitivity analysis (PSA) was conducted using MCS with 10 000 iterations. Utility values, disease prevalence and transition probabilities were modelled using a beta distribution, while the cost parameters used a gamma distribution. The intervention effect on the knowledge index and the diagnostic performance of the screening tool was modelled using a normal distribution. Relative risk or HR was modelled using a log-distribution. Additionally, a one-way sensitivity analysis was performed by varying each parameter individually to its CI when available or ±30% of its base case value, while keeping other parameters constant.^35^ Both one-way sensitivity and scenario analyses were conducted using a fixed set of 10 000 MCS iterations from the first simulation, ensuring that observed changes in costs and QALYs reflect only the intended parameter variations and not random noise from different simulations. Information about the distribution and source of each input parameter is further explained in online supplemental table E1. Input Parameters.

### Patient and public involvement

This study is part of the broader SUNI-SEA project, which includes partners from Universitas Sebelas Maret Indonesia. The project engaged a wide range of stakeholders, such as researchers, healthcare staff, patients, policymakers and national and local health authorities. Specifically for this research, their input contributed to both the clinical validity of the model and the validity of the key assumptions, ensuring that the assumptions made in the model closely aligned with the actual situation.

## Results

### Cost-effectiveness of one-time CBHI strategies

Compared with the reference scenario, all one-time CBHI strategies led to higher expected QALYs and total costs, with the combined health education and blood glucose plus NCD risk factor screening yielding the highest outcomes (21 465.89 QALYs and Int$2 685 457.74 for the reference scenario; 21 467.37 QALYs and Int$2 698 218.14 for screening only; and 21 469.96 QALYs and Int$2 699 912.96 for the combined strategy). Using a cost-effectiveness threshold of one GDP per capita in Indonesia (Int$14 445.70), both strategies were considered cost-effective compared with the reference scenario, with ICERs of Int$3550.28/QALY gained for the combined strategy and Int$8597.46/QALY gained for the screening-only strategy (table 3).

**Table 3:** Cost-effectiveness results of different CBHI strategies

	Reference scenario	T2D screening	T2D screening and health education
Cost of CBHI Int$ (SE)			
One-time CBHI	0	1997.20 (2.45)	7056.97 (7.97)
Repeated CBHI	–	10 476.47 (14.26)	15 978.14 (16.10)
Cost of treatment Int$ (SE)			
One-time CBHI	2 685 457.74 (9655.36)	2 696 220.94 (9676.00)	2 692 855.99 (9662.95)
Repeated CBHI	–	2 701 209.29 (9685.73)	2 698 173.24 (9673.95)
Total cost Int$ (SE)			
One-time CBHI	2 685 457.74 (9655.36)	2 698 218.14 (9677.24)	2 699 912.96 (9664.09)
Repeated CBHI	–	2 711 685.76 (9687.64)	2 714 151.38 (9675.75)
Total QALYs gained (SE)			
One-time CBHI	21 465.89 (63.55)	21 467.37 (63.55)	21 469.96 (63.56)
Repeated CBHI	–	21 468.18 (63.55)	21 470.92 (63.56)
Incremental cost Int$ (SE)			
One-time CBHI	–	12 760.41 (45.23)	14 455.22 (102.81)
Repeated CBHI	–	26 228.02 (67.95)	28 693.65 (100.99)
Incremental QALYs gained (SE)			
One-time CBHI	–	1.48 (0.01)	4.07 (0.07)
Repeated CBHI	–	2.29 (0.01)	5.03 (0.08)
ICER (Int$/QALY gained)			
One-time CBHI	–	8597.46	3550.28
Repeated CBHI	–	11 461.91	5699.76

Reference scenario: no T2D screening nor health education at the community level.

CBHI, community-based health intervention; ICER, incremental cost-effectiveness ratio; Int$, international dollars; QALYs, quality-adjusted life years; T2D, type 2 diabetes mellitus.

### Scenario analysis

When the CBHI scenarios included repeated annual screening for pre-diabetes, moderate or high-risk individuals, as well as screening every 3 years for low-risk individuals, all CBHI scenarios resulted in higher expected QALYs gained and total cost compared with the one-time CBHI strategy (21 468.18 QALYs and Int$2 711 685.76 for repeated screening only and 21 470.92 QALYs and Int$2 714 151.38 for repeated screening and health education). Compared with the reference scenario, the repeated CBHI strategies remained cost-effective within the threshold of one GDP per capita in Indonesia (ICER of Int$11 461.91/QALY gained for screening only and Int$5699.76/QALY gained for screening with health education) (table 3).

By varying the uptake rates of CBHI and adherence to PHC referral recommendations, we found that a minimum of a 10% rate for uptake or adherence to follow-up tests at PHC is needed for a repeated screening-only scenario to be cost-effective under the one GDP per capita threshold. Additionally, improving the rates of follow-up for diagnostic tests and treatment at PHC would make both one-time and repeated screening-only strategies more cost-effective than solely improving their uptake level (online supplemental figure F1-F4. Scenario Analysis).

Online supplemental figure F5 shows the minimum knowledge gain required to improve the lifestyle index and make the intervention cost-saving. Specifically, if the knowledge gain increased to 3.4 points (out of a potential 18-point increase), community-level health education could potentially improve the lifestyle index among high-risk individuals, making the intervention cost-saving.

### Sensitivity analysis

**Figure 1:**
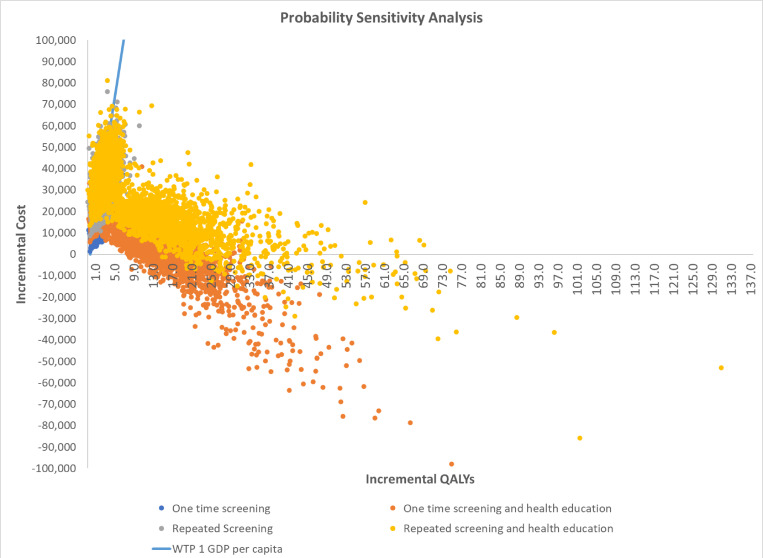
Probabilistic sensitivity analysis. GDP, gross domestic product; QALYs, quality-adjusted life years; WTP 1, Willingness to Pay 1.

The PSA cost-effectiveness plane and the cost-effectiveness acceptability curves of different CBHI strategies are presented in figures1  2, respectively. Overall, most of the simulations are clustered in the northeast and southeast quadrants, indicating that all CBHI scenarios were likely to be cost-effective or cost-saving, compared with the reference scenario. However, considerable uncertainty remains. Within the threshold of one GPD per capita, one-time and repeated screening alone had 88.0% and 70.8% probability of being cost-effective, respectively. In contrast, one-time and repeated screenings with health education had only a 72.1% and 60.0% probability of being cost-effective, respectively. When the threshold was increased to three GPD per capita, the likelihood of all CBHI strategies being cost-effective rose to 99.7%, 99.0%, 98.9% and 98.3% for the scenario of one-time and repeated screening without and with health education, respectively.

**Figure 2:**
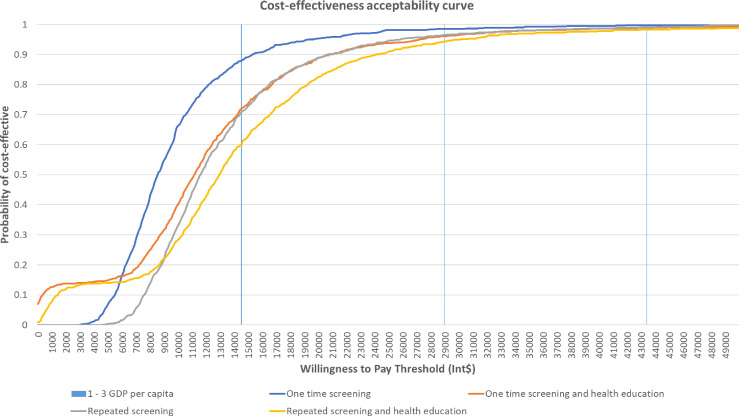
Cost-effectiveness acceptability curve. GDP, gross domestic product; Int$, international dollars.

The results of the one-way sensitivity analyses for one-time and repeated CBHI strategies without or with health education are provided in online supplemental figures G1-G4. One-way Sensitivity Analysis.

In general, one of the main contributors to uncertainty surrounding the expected ICER value of all CBHI strategies was the risk of CVD incidence among undiagnosed patients with T2D. When the risk was lower than what we assumed for the main model input parameter, it led to lower expected incremental QALYs gained. Consequently, all scenarios of screening-only strategies were not cost-effective under one GDP per capita in Indonesia (ICER one-time: Int$21 631.33/QALY gained and repeated: Int$26 978.23/QALY gained), and those involving health education became costlier (one-time only: Int$5841.72/QALY gained and repeated: Int$9348.98/QALY gained).

Other main parameters that influenced the cost-effectiveness of one-time screening-only strategies were the sensitivity of the screening tool for detecting T2D, the utility value of undiagnosed T2D and T2D treated at the PHC level, and the cost of T2D treatment at the PHC level. When the sensitivity was lower, it resulted in lower health gain and a higher total cost, as the number of missed detected T2D cases increased. Consequently, the screening-only strategy became costlier at Int$10 259.17/QALY gained. When the utility value for patients with T2D treated at the PHC was lower or the treatment cost at the PHC was higher, it resulted in an increased cost per QALY gained, to Int$10 514.15 and Int$10 469.86, respectively. Likewise, a higher utility value for undiagnosed diabetes made the screening-only scenario costlier at Int$9872.27/QALY gained. Despite these changes, the one-time screening-only strategy was still deemed cost-effective under the threshold of one GDP per capita in Indonesia.

For a one-time screening and health education scenario, risk reduction in pre-diabetes or T2D incidence due to lifestyle improvement, the effect of CBHI on the knowledge improvement, and baseline lifestyle index among the high-risk T2D group were contributors to uncertainties. When the baseline lifestyle index among high-risk populations was higher, while the risk reduction and effect of CBHI on knowledge improvement were lower, it resulted in lower health gain, higher total cost and higher ICER values (ICER ranges from Int$5543.76 to Int$9109.66 per QALY gained).

When the two scenarios were repeated to include re-screening low-risk individuals every 3 years or annually for pre-diabetes, moderate and high-risk individuals, the main parameter that led to the uncertainty was the size of the population that one CBHI could serve. If the average population per province were lower, it would result in a lower number of target populations per village, leading to lower health gain. Consequently, the repeated screening scenario would not be cost-effective under one GDP per capita threshold, with an ICER of Int$15 166.55 per QALY gained. For strategies involving health education, it became costlier to $Int8356.69 per QALY gained.

## Discussion

This study assessed the cost-effectiveness of community-level screening for T2D and its risk factors, either alone or with health education, compared with no intervention. Our findings indicate that, despite the currently low uptake level of 10.6%, which was also affected by the COVID-19 restrictions, all CBHI strategies offering screening only are cost-effective within the threshold of one GDP per capita in Indonesia. The strategy becomes more cost-effective when combined with health education, which potentially leads to cost savings if it effectively promotes higher knowledge gain. This highlights the need to ensure effective knowledge transfer before scaling up CBHI’s coverage. Additionally, scenario analysis revealed that enhancing coordination with PHC, that is, increasing referral adherence for confirmatory testing and treatment, results in lower costs per QALY gained compared with merely improving uptake levels for a screening-only strategy. Nevertheless, uncertainty remains, particularly for screening-only strategies where the cost-effectiveness estimates depend on the risk of complications among the undiagnosed patients with T2D.

To the best of our knowledge, no study has explored the cost-effectiveness of CBHI, which combines T2D screening and health education. Nevertheless, our findings are consistent with a previous health economic evaluation in Vietnam, which found that providing one-time T2D screening at the community level is cost-effective from a payer perspective and dominates screening at the PHC when it is repeated every 3 years.^14^ Similarly, a study in Indonesia reported that community-level T2D screening using RCBG incurred a higher cost but lower disability-adjusted life years lost compared with PHC-level screening (7.10 vs 7.11 DALYs lost, respectively).^15^

Our analysis also indicated that increasing CBHI’s uptake would enhance cost-effectiveness. This finding contrasts with a study in India, which found that population-based T2D and hypertension screening was not cost-effective unless more than 20% of newly diagnosed cases were treated at the PHC level.^52^ The discrepancy may be due to differences in disease patterns, with the prevalence of diabetes among individuals over 35 years old in Indonesia being nearly three times higher than in India (10.1% vs 3.4%, respectively).^52 53^ Nonetheless, our scenario analysis supports the Indian study’s conclusion that improving coordination with PHC for managing newly detected T2D cases could result in a lower cost per QALY gained compared with solely expanding the community-based T2D screening coverage.

Since 2013, the Indonesian government has implemented an Integrated Health Services (*Pandu PTM*) policy to control the burden of NCDs. A central component of this policy is to diagnose probable T2D cases detected through CBHI and treat uncomplicated T2D and hypertension cases through the Chronic Disease Management (*Prolanis*) programme at the PHC level.^9^ A recent study estimated that *Prolanis* coverage increased from less than 5% in 2014 to almost 50% in 2016. However, coverage remains lower in areas outside of Java Island.^34^ Therefore, it is imperative to address disparities in *Prolanis* coverage to ensure the cost-effectiveness of CBHI, particularly when planning to expand CBHI coverage nationally.

Nevertheless, our cost-effectiveness estimates of the screening-only strategies are sensitive to the quality of life value for patients with T2D treated at the PHC level. This suggests that managing newly detected diabetes cases at the PHC level, as opposed to the secondary care level in the reference scenario, could become costlier if PHC-level treatment is ineffective in controlling the disease. However, previous studies have highlighted the suboptimal PHC readiness for NCD care and the suboptimal improvement in clinical outcomes of patients with T2D treated at the PHC level through the *Prolanis* programme.^54^,^56^ Therefore, ensuring PHC readiness and improving the quality of *Prolanis* to deliver NCD care are essential for ensuring the cost-effectiveness of CBHI implementation in Indonesia.

Additionally, our sensitivity analysis found that a screening-only strategy may not be cost-effective under the threshold of one GDP per capita in Indonesia when the risk of diabetes complications among the undiagnosed group is lower than assumed in the base case scenario. However, this estimate may be underestimated, as we only included CVD as a proxy for complications. Generally, microvascular complications occur more frequently than macrovascular events, even among newly diagnosed T2D cases.^57 58^ Furthermore, the presence of macrovascular disease is a strong predictor of future microvascular complications, contributing to poorer outcomes, reduced quality of life and higher healthcare costs.^5 49 58^ Including a broader range of complications in the model may yield more robust estimates and strengthen the conclusion that T2D screening is a cost-effective strategy.

We demonstrated that incorporating a health education component alongside community-level T2D screening resulted in a higher expected health gain, thereby making the intervention more cost-effective. Nevertheless, given the current level of health education effectiveness, there is considerable uncertainty surrounding the cost-effectiveness estimates, particularly on how this knowledge improvement would result in the adoption of a new healthy lifestyle. Similarly, a prior study of community-based low-intensity lifestyle interventions across Europe found that, although the overall intervention was cost-effective, its effectiveness among the high-risk group was uncertain. This uncertainty was likely due to the minimal impact of the intervention on behavioural changes in this group.^23^

Our scenario analysis also indicated that community-based T2D screening combined with health education could lead to greater health gains and make the intervention cost-saving, provided that health education increases T2D knowledge by at least 3.4 points (out of 18 points maximal improvement) compared with non-CBHI participants. These findings align with a recent simulation study in Indonesia, which showed that expanding T2D screening along with effective risk factor control interventions could prevent up to 42.6% of new T2D cases, compared with only 5.5% by extending screening coverage alone.^7^ In our study, we observed only a 0.86-point improvement in the knowledge index, possibly reflecting an underestimated CBHI effect, as our analysis did not account for potential community-level spillover effects reported in prior systematic reviews on other health conditions.^59^

Our cost-effectiveness model has several strengths. First, it relies on local data, providing a realistic depiction of CBHI implementation in Indonesia. Second, the model adopts a pragmatic approach by including potential additional costs from false positives and false negatives in T2D screening, providing a more accurate representation of the screening programme’s effectiveness. Third, it accounts for a cascade of care by considering the likelihood of loss to follow-up after screening, thereby making our estimates more conservative. Finally, the model includes programme costs, encompassing start-up expenses for developing health education materials, training and remuneration for community health workers, which have been noted to improve community health workers’ satisfaction and therefore are crucial to ensure the quality of CBHI delivery.^60^

However, our study has certain limitations. First, we only used CVD as a proxy for T2D complications, as it was the only available longitudinal data on diabetes complications in Indonesia. Although this may underestimate the result, CVD is the most common and costly diabetes complication in the country.^5^ As such, our estimates still reflect a substantial part of CBHI’s potential cost-effectiveness. Second, we assessed the effectiveness of health education solely based on individual-level knowledge improvement, assuming a linear relationship between knowledge improvement and behavioural change.^61^ However, recent evidence suggests that environmental factors also significantly influence behavioural changes, which could lead to an overestimation of our results.^62 63^ Additionally, we did not account for the possible spillover effect at the community level, which may also result in underestimated cost-effectiveness estimates. Third, we estimated the diagnostic performance of RCBG for T2D screening using data from India, where the screening cut-off value is lower than in Indonesia (140 mg/dL vs 200 mg/dL).^43^ This discrepancy may have led to an overestimation of the specificity value. Fourth, we did not include societal costs, as recommended in the guidelines.^21 32^ Including these costs could have improved cost-effectiveness estimates, as highlighted by a prior study from the USA, which showed a high indirect cost, such as labour productivity losses, associated with T2D conditions.^64^ Fifth, we did not account for the distributional effect of CBHI utilisation across different sociodemographic groups, whereas evidence has pointed out a significant underutilisation of such service among workers and the male population.^65^ Lastly, the results should be interpreted with caution as they primarily apply to settings with similar epidemiological patterns of T2D and its associated risk factors to those in Indonesia.

Looking forward, it is imperative to evaluate the effectiveness of CBHI in reducing T2D risk factors at both the individual and community levels, to truly gauge its potential impact on lowering T2D incidence. In particular, the added value of knowledge-enhancing interventions and their role in facilitating behavioural change, along with their possible spillover effect. Further research could also explore the effectiveness of delivering health education through modern communication methods, such as social media and e-health tools, that have been initiated by the SUNI-SEA project.^66^ Additionally, given the influence of screening tool performance on the cost-effectiveness estimates, further research is needed to determine the optimal cut-off value for RCBG for the Indonesian population. Further health economic analysis could also compare different screening modalities, such as FINDRISC (Finnish Diabetes Risk Score) and ADA (American Diabetes Association) diabetes risk scores, which have been validated in Indonesia, while also incorporating the distributional impact of such interventions across sociodemographic groups.^67 68^ On top of these suggestions, our findings underscore the importance of effective T2D management at the PHC level and the need for effective community-level health education methods. Therefore, before expanding CBHI strategies, it is essential to assess and enhance the quality, along with the readiness of PHC services for T2D care. Ensuring adequate training for community health workers is also needed to improve the quality of health education and overall CBHI programme delivery.

## Conclusions

Our study suggests that, given the current level of uptake and adherence to PHC referral recommendations, community-based screening for T2D with or without health education is cost-effective. The strategies could become more cost-effective or cost-saving with better coordination with PHCs in managing newly diagnosed patients with T2D and enhanced health education effectiveness. Nonetheless, resource allocators must ensure that PHCs have the capacity and resources to manage newly diagnosed or high-risk T2D cases before scaling up CBHI to a wider area.

## Supplementary material

10.1136/bmjph-2024-002161 online supplemental file 1

## Data Availability

Data are available upon reasonable request.
