# Supplementary material for: Cost-effectiveness of community-based type 2 diabetes prevention and control in Indonesia: a health economics modelling study
Source: BMJ Public Health. 2025 Oct 7;3(2):e002161. doi: 10.1136/bmjph-2024-002161 (PMC12506045; doi:10.1136/bmjph-2024-002161)
Supplement: online supplemental file 1 [file bmjph-3-2-s001.docx]

Supplementary Material: Cost-Effectiveness of Community-Based Type 2 Diabetes Prevention and Control in Indonesia: A Health Economics Modelling Study

[A. Summary of the SUNI SEA Project 2](#_Toc202348183)

[B. Illustration of the Decision Tree and Markov Model 4](#_Toc202348184)

[C. Effect of Posyandu Participation on Knowledge Level 6](#_Toc202348185)

[D. Program and Intervention Cost to Establish One Community-Based Health Intervention 10](#_Toc202348186)

[E. Input parameters 11](#_Toc202348187)

[F. Scenario Analysis 24](#_Toc202348188)

[G. One-way sensitivity analysis 29](#_Toc202348189)

[References 37](#_Toc202348191)

# Summary of the SUNI SEA Project

“Scaling-up Non-communicable disease (NCDs) interventions in Southeast Asia” (SUNI-SEA) was a four-year research project aimed at evaluating and validating effective and cost-effective scaling-up strategies for preventing and controlling NCDs in Indonesia, Vietnam, and Myanmar. In Indonesia, the project focused on assessing the coverage and effectiveness of the community-based health intervention (CBHI) *Pos Pembinaan Terpadu Penyakit Tidak Menular (Posbindu),* which has now been merged with other CBHI initiatives in the country and rebranded under the name of *Pos Pelayanan Terpadu* (*Posyandu).*[1] Despite the rebranding, the intervention continues to aim at preventing and controlling the burden of NCDs, including Type 2 Diabetes Mellitus (T2D), through NCD risk factor and blood glucose screening, health education, and referral to the disease management program at the primary health care (PHC) level.[2]

The SUNI-SEA baseline survey in Indonesia was conducted between August and December 2021 in two cities (Surakarta and Kediri) and two regencies (Batang and Jember). The sample was selected using a multistage sampling method. First, five PHCs *(Puskesmas)* were randomly selected in each study area. From each of the Puskesmas, three *Posyandu* centres were randomly selected, leading to a total of 60 *Posyandu.* From each *Posyandu*, ten individuals from the participant list were randomly selected. Additionally, ten households within the target area of each *Posyandu* were randomly selected, with two members from each household being interviewed for the survey (Figure A1). The inclusion criteria for the households were that they must reside in close proximity to *Posyandu* and have at least one member older than 15 years old. In total, 1,800 individuals were interviewed during the baseline survey, comprising 600 individuals from the *Posyandu* sample and 1,200 individuals from the household sample.

The clinical trial number of the SUNI-SEA project is NCT05239572.

**Figure A1: Sampling strategy within the SUNI-SEA project**

# Illustration of the Decision Tree and Markov Model


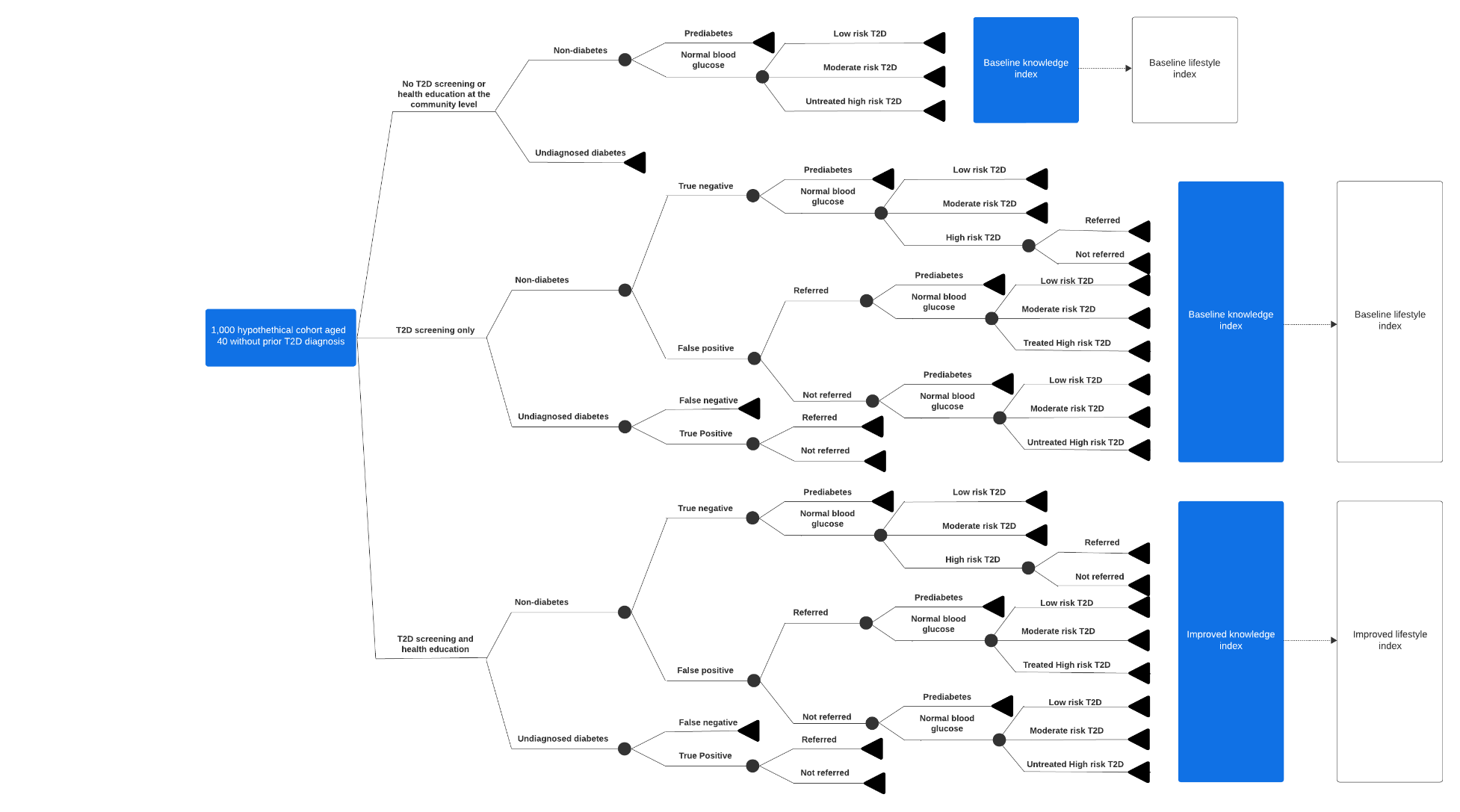


**Figure B1 Decision Tree of Different CBHI Strategies**

**
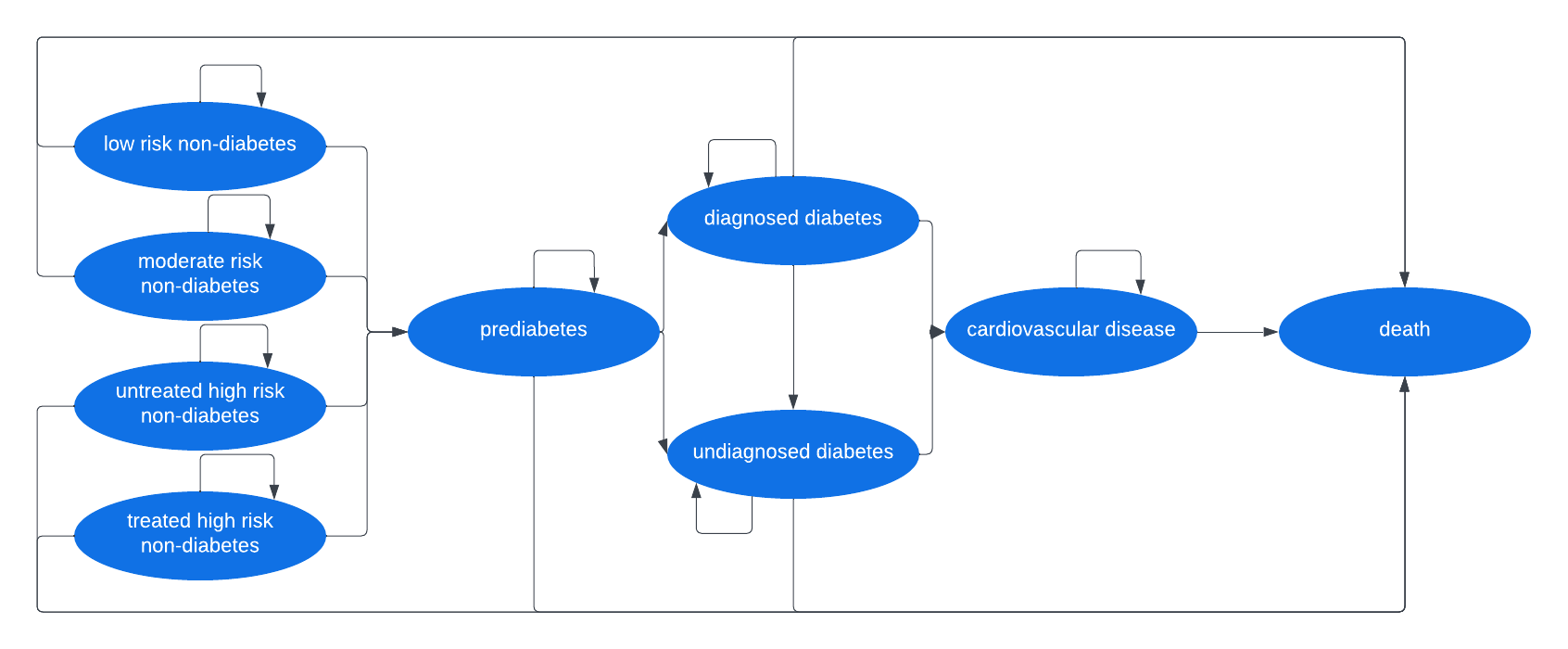
**

**Figure B2 Markov Model to Extrapolate the Output of the Decision Tree**

# Effect of Posyandu Participation on Knowledge Level

We employed propensity score matching (PSM) to assess the effect of *Posyandu* participation on the improvement in T2D knowledge levels by estimating the conditional likelihood of the involvement for each member of the sample through empirical modelling based on observable variables gathered from the baseline survey. The sample used for the PSM comprised all participants from the baseline survey aged 40 and older who did not have a self-reported diagnosis of diabetes and had complete data on all variables used in the matching algorithm, resulting in a total sample of 1,030 individuals. The following variables were used to create the propensity score: age, gender, asset index, type of occupation, level of education, district of residence, household income, age of the household head, travel time to the nearest Primary Health Care (PHC), history of blood pressure diagnosis by doctor frequency to visit *PHC* in the past year, and the number of NCD related symptoms that occurred in the last 6 months. The logit specification for estimating the propensity score matching and the difference of covariates between *Posyandu* and *non-Posyandu* participants before and after matching are displayed in Tables C1 and C2, respectively.

After matching, there is no significant differences between participation vs no participation in *Posyandu,* except for the marital status of “separated/ divorced/ widowed” category.

**Table C1 Logit Specification for Propensity Score**

| **Variable** | **Coefficient** | **SE** | **P value** |
| --- | --- | --- | --- |
| Age | -0.009 | 0.170 | 0.558 |
| Gender (ref: female) | -1.301 | 0.176 | 0.000 |
| Asset index | -0.025 | 0.065 | 0.704 |
| Marital status (ref: never married) |  |  |  |
| Currently married | -0.050 | 0.768 | 0.951 |
| Separated/ divorced/ widowed | -0.047 | 0.768 | 0.951 |
| Education (ref: no formal schooling) |  |  |  |
| Elementary school | 0.736 | 0.389 | 0.058 |
| High school | 0.749 | 0.414 | 0.070 |
| Adult education (A/B/C) and university | 0.869 | 0.481 | 0.071 |
| District (ref: Batang) |  |  |  |
| Surakarta | 0.170 | 0.221 | 0.441 |
| Jember | -0.437 | 0.231 | 0.058 |
| Kediri | -0.185 | 0.225 | 0.410 |
| Income (ref: No income) |  |  |  |
| 0 - < 1 million IDR | -0.074 | 0.207 | 0.719 |
| 1 - < 2 million IDR | -0.097 | 0.228 | 0.670 |
| 2 - < 5 million IDR | -0.657 | 0.387 | 0.090 |
| Age of head of the household | 0.051 | 0.014 | 0.000 |
| Travel time to the nearest PHC | 0.046 | 0.012 | 0.000 |
| Prior hypertension diagnosis by doctor (ref: no) | 0.024 | 0.223 | 0.916 |
| Visited *Puskesmas* in the past 6 months | 0.079 | 0.051 | 0.122 |
| Number of NCDs related symptoms in the past 6 months | 0.200 | 0.142 | 0.157 |
| Occupation (ref: farmer) |  |  |  |
| Working in non-agricultural sector | -0.006 | 0.276 | 0.983 |
| Retired/ old/ sick/ not working | -0.064 | 0.276 | 0.838 |
| Housewife | -0.524 | 0.316 | 0.090 |
| Other | 0.081 | 0.309 | 0.815 |
| **Number of observations** | 1.030 |  |  |
| **Pseudo R-squared** | 0.1674 |  |  |
| **Log likelihood** | -591.936 |  |  |

**Table C2 Balance of baseline covariates – unmatched and matched sample**

| **Variables** | **Unmatched Samples** | | | **Matched Samples** | | |
| --- | --- | --- | --- | --- | --- | --- |
|  | **Control**  (N= 476) | **Treatment**  (N= 554) | **P-value** | **Control**  (N= 476) | **Treatment**  (N= 542) | **P-value** |
| Age | 50.027 | 51.991 | <0.001 | 51.779 | 50.928 | 0.113 |
| Gender (ref: female) | 0.540 | 0.193 | <0.001 | 0.197 | 0.199 | 0.939 |
| Asset index | 4.661 | 4.442 | 0.084 | 4.459 | 4.421 | 0.651 |
| Marital status (ref: never married) | 0.008 | 0.014 | 0.369 | 0.013 | 0.017 | 0.615 |
| Currently married | 0.926 | 0.790 | <0.001 | 0.806 | 0.851 | 0.053 |
| Separated/ divorced/ widowed | 0.065 | 0.195 | 0.000 | 0.181 | 0.133 | 0.030 |
| Education (ref: no formal schooling) | 0.042 | 0.038 | 0.737 | 0.037 | 0.050 | 0.297 |
| Elementary school | 0.313 | 0.368 | 0.063 | 0.365 | 0.327 | 0.180 |
| High school | 0.540 | 0.495 | 0.147 | 0.496 | 0.530 | 0.274 |
| Adult education (A/B/C) and university | 0.105 | 0.099 | 0.761 | 0.101 | 0.094 | 0.683 |
| District (ref: Batang) | 0.227 | 0.231 | 0.874 | 0.234 | 0.234 | 1.000 |
| Surakarta | 0.229 | 0.309 | 0.004 | 0.303 | 0.286 | 0.549 |
| Jember | 0.258 | 0.231 | 0.308 | 0.232 | 0.220 | 0.612 |
| Kediri | 0.286 | 0.229 | 0.038 | 0.231 | 0.260 | 0.259 |
| Income (ref: No income) | 0.206 | 0.280 | 0.006 | 0.273 | 0.306 | 0.228 |
| 0 - < 1 million IDR | 0.374 | 0.336 | 0.201 | 0.334 | 0.295 | 0.170 |
| 1 - < 2 million IDR | 0.353 | 0.341 | 0.692 | 0.349 | 0.349 | 1.000 |
| 2 - < 5 million IDR | 0.067 | 0.043 | 0.092 | 0.044 | 0.050 | 0.667 |
| Age of head of the household | 51.420 | 55.440 | <0.001 | 55.173 | 55.133 | 0.943 |
| Travel time to the nearest PHC | 10.853 | 11.866 | 0.012 | 11.797 | 12.330 | 0.913 |
| Prior hypertension diagnosis by doctor (ref: no) | 0.160 | 0.255 | <0.001 | 0.247 | 0.245 | 0.944 |
| Visited *Puskesmas* in the past 6 months | 0.600 | 0.995 | <0.001 | 0.932 | 1.066 | 0.213 |
| Number of NCDs related symptoms in the past 6 months | 0.292 | 0.484 | <0.001 | 0.463 | 0.487 | 0.563 |
| Occupation (ref: farmer) | 0.137 | 0.085 | 0.008 | 0.087 | 0.078 | 0.581 |
| Working in non-agricultural sector | 0.534 | 0.404 | <0.001 | 0.408 | 0.376 | 0.291 |
| Retired/ old/ sick/ not working | 0.109 | 0.132 | 0.270 | 0.133 | 0.144 | 0.598 |
| Housewife | 0.132 | 0.314 | <0.001 | 0.306 | 0.327 | 0.473 |
| Other | 0.086 | 0.065 | 0.199 | 0.066 | 0.076 | 0.555 |
| Ps R2 | 0.014 |  |  |  |  |  |
| LR chi2 | 20.39 |  |  |  |  |  |
| Mean Bias | 5.3 |  |  |  |  |  |
| B | 27.5 |  |  |  |  |  |


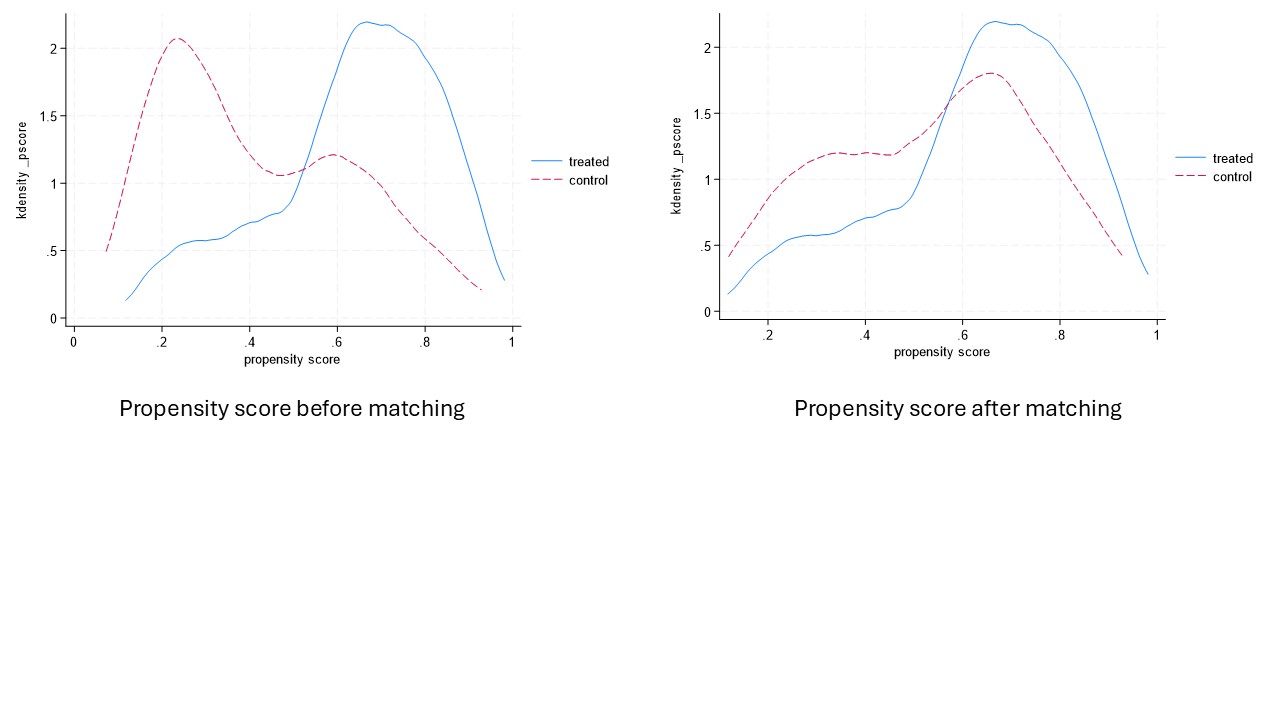


**Figure C1 Kernel density distributions of propensity scores before and after matching**

The T2D knowledge index score was measured from the following questions in which each of the correct answer were assigned one point as a score:

1. Type 2 Diabetes Mellitus (T2D) is a disease in which there is an increase in blood sugar levels above normal
2. If a person has a healthy diet, exercising regularly is not necessary for preventing chronic diseases such as T2D or hypertension
3. Eating vegetables is harmful if someone has T2D
4. Only overweight people can get T2D
5. Diabetes can be contagious via the exchange of blood or liquids
6. You can feel normal and healthy but still have T2D without noticing it
7. Herbal medicines compared to medicines prescribed by a doctor are better to control chronic diseases

Additionally, the respondents were asked to independently identify the risk factors, symptoms, and complications of diabetes, without being provided with a list of answer options. Each correct response was awarded one point:

1. What are the risk factors of T2D?
   1. Obesity/ overweight
   2. Physical inactivity
   3. Older age
   4. High sugar consumption
   5. Family history of diabetes
   6. History of high blood pressure
2. What are the symptoms of T2D?
   1. Increased thirst
   2. Frequent urination
   3. Increased hunger
   4. Dry mouth
   5. Blurred vision
   6. Unexplained weight loss
   7. Fatigue
   8. Headaches
3. What are the complications of T2D?
   1. Dental and gum disease
   2. Eye problems and sight loss
   3. Foot damage/ foot problems
   4. Heart diseases
   5. Nerve damage
   6. Stroke
   7. Kidney disease

The total score of T2D knowledge ranged from 0 – 28.

# Program and Intervention Cost to Establish One Community-Based Health Intervention

| Item | Unit | Frequency | Average Cost (IDR 2022) | Average Cost (Int$ 2022) |
| --- | --- | --- | --- | --- |
| Community health workers training | Per community* | One time | 2,691,666 | 554.90 |
| Community health workers incentive | Per community* | Once a year | 4,650,000 | 958.62 |
| RCBG kit | Per individual screened | Once a year | 20,000 | 4.12 |
| *Posyandu* Application development** | Per application | One time | 25,000,000 | 5,153.85 |
| Health education material development | Per community | One time | 284,055.56 | 58.56 |

**Notes:**

*CBHI runs once a month and is delivered by five community health workers.

***Posyandu* application was developed as a tool to deliver health education.

# Input parameters

| **Input Parameters** | **Mean** | **SE or CI** | **Distribution** | **Description** | **Sources** |
| --- | --- | --- | --- | --- | --- |
| **Decision Tree** |  |  |  |  |  |
| Prevalence of undiagnosed T2D | 0.091 | 0.080 – 0.104 | Beta | Prevalence of undiagnosed T2D among people aged 35 – 45 years old in Indonesia. Calculated by subtracting the total diabetes prevalence with the prevalence of diabetes based on self-reported doctor diagnosis among the group (10.1% vs 1%, respectively). | [3] |
| Prevalence of moderate risk non-diabetic | 0.129 | 0.092 – 0.140 | Beta | Percentage of people aged ≥ 40 years without a prior diabetes diagnosis who were categorized as moderate risk for T2D, according to the criteria stated in Table 1 of the main text. | SUNI-SEA Baseline Survey among household samples aged ≥ 40 years without prior diabetes diagnosis |
| Prevalence of high risk non-diabetic | 0.757 | 0.723 – 0.789 | Beta | Percentage of people aged ≥ 40 years without a prior diabetes diagnosis who were categorized as high risk for T2D, according to the criteria stated in Table 1 of the main text. | SUNI-SEA Baseline Survey among household samples aged ≥ 40 years old without prior diabetes diagnosis |
| Sensitivity of RCBG to detect T2D | 0.786 | 0.550 – 1.000 | Beta | Estimated from the sensitivity of RCBG for detecting diabetes with a cut-off value of 140 mg/dL | [4] |
| Specificity of RCBG to detect T2D | 0.839 | 0.587 – 1.000 | Beta | Estimated from the specificity of RCBG for detecting diabetes with a cut-off value of 140 mg/dL | [4] |
| Adherence to repeated screening | 0.060 | 0.042 – 0.078 | Beta | Estimated adherence rate to follow re-screening recommendations. The value is assumed to be similar to the rate of adherence to the PHC referral recommendation. | SUNI-SEA Baseline Survey |
| Uptake of CBHI | 0.107 | 0.090 – 0.126 | Beta | Percentage of individuals who reported visiting *Posyandu* in the past 12 months. | SUNI-SEA Baseline Survey |
| Adherence to the PHC referral recommendation | 0.060 | 0.042 – 0.078 | Beta | Percentage of individuals who reported visiting PHC after being referred by community health workers during *Posyandu* activity | SUNI-SEA Baseline Survey |
| **Markov model** |  |  |  |  |  |
| Transition probability from non-diabetes to prediabetes | 0.030 | 0.026 – 0.033 | Beta | Yearly incidence rate of prediabetes among non-glucose tolerant in the CURES study. | [5] |
| Transition probability from non-diabetes to death | Age-standardized mortality rate | | | | [6] |
| Transition probability from prediabetes to diagnosed T2D | 0.021 | Standard errors and probability distributions were only applied to the individual parameters used to estimate this input parameter. | | Estimated by multiplying the yearly incidence of diabetes among the prediabetes population from the CURES study by the estimated proportion of diagnosed diabetes cases within the overall diabetes prevalence in Indonesia. | [5, 7] |
| Transition probability from prediabetes to undiagnosed T2D | 0.058 | Standard errors and probability distributions were only applied to the individual parameters used to estimate this input parameter. | | Estimated by multiplying the yearly incidence of diabetes among the prediabetes population from the CURES study by the estimated proportion of undiagnosed diabetes cases within the overall diabetes prevalence in Indonesia. | [5, 7] |
| Transition probability from prediabetes to death | Age-standardized mortality rate | | | | [6] |
| Transition probability from T2D to CVD | 0.008 | 0.005 – 0.010 | Beta | Yearly incidence rate of CVD among dysglycemic patient from the Bogor Cohort Study.  Recalculated from the 6-year incidence rate of CVD events with the value of 4.5%.* | [8] |
| Transition probability from diabetes to death | Age-standardized mortality rate multiplied by diabetes mortality risk | | | | [6, 9] |
| Transition probability from undiagnosed T2D to CVD | 0.020 | Standard errors and probability distributions were only applied to the individual parameters used to estimate this input parameter. | | Estimated by multiplying the yearly incidence rate of CVD among dysglycemic population in the Bogor Cohort Study by the risk of CVD event among undiagnosed diabetes.* | [8] |
| Transition probability from undiagnosed T2D to diagnosed T2D | 0.0004 | Standard errors and probability distributions were only applied to the individual parameters used to estimate this input parameter. | | Estimated from the increase in the prevalence of self-reported diagnosed diabetes among the general population between 2018 and 2023. Assuming a constant detection rate, the value was recalculated into a yearly probability.* | [3, 10] |
| Transition probability from undiagnosed T2D to death | Age standardized mortality rate multiplied by risk of mortality among T2D and the risk of CVD event among undiagnosed T2D.* | | | | [6, 8, 9] |
| Transition probability from CVD to death | Age standardized mortality rate multiplied by risk of mortality among CVD* | | | | [6, 9] |
| **Baseline parameters** |  |  |  |  |  |
| Baseline knowledge level of low-risk non-diabetic group | 8.300 | 4.940 – 11.660 | Normal | Average knowledge level of non-CBHI participants categorized as low-risk for T2D | SUNI-SEA Baseline Survey |
| Baseline knowledge level of moderate-risk non-diabetic group | 8.790 | 5.630 – 11.950 | Normal | The average knowledge level of non-CBHI participants categorized as moderate-risk for diabetes | SUNI-SEA Baseline Survey |
| Baseline knowledge level of high-risk non-diabetic group | 9.340 | 6.270 – 12.410 | Normal | The average knowledge level of non-CBHI participants categorized as high-risk for diabetes | SUNI-SEA Baseline Survey |
| Baseline knowledge level of prediabetes group | 8.790 | 5.630 – 11.950 | Normal | The value was assumed to be similar to non-CBHI participants categorized as moderate-risk of diabetes | Assumption |
| Baseline lifestyle index of low-risk non-diabetic group | 2.156 | 1.646 – 2.666 | Normal | Average lifestyle index of non-CBHI participants categorized as low-risk for diabetes | SUNI-SEA Baseline Survey |
| Baseline lifestyle index of moderate-risk non-diabetic group | 2.096 | 1.266 – 2.926 | Normal | Average lifestyle index of non-CBHI participants categorized as moderate-risk for diabetes | SUNI-SEA Baseline Survey |
| Baseline lifestyle index of high-risk non-diabetic group | 2.380 | 1.680 – 3.080 | Normal | Average lifestyle index of non-CBHI participants categorized as high-risk for diabetes | SUNI-SEA Baseline Survey |
| Baseline lifestyle index of the prediabetes group | 2.096 | 1.266 – 2.926 | Normal | The value was assumed to be similar with non-CBHI participants categorized as moderate-risk of diabetes | Assumption |
| **Multiplier** |  |  |  |  |  |
| Association between knowledge and lifestyle index | 0.073 | 0.053 – 0.092 | Normal | Beta coefficient of linear regression between knowledge level and lifestyle index. | SUNI-SEA Baseline Survey |
| Risk of prediabetes incidence among the high-risk non-diabetic group | 1.030 | 1.000 – 1.050 | Lognormal | Estimated from the HR of dysglycemia incidence among the population who has central obesity in the CURES study. | [5] |
| Risk of prediabetes incidence among individuals having 2 lifestyle behaviours. | 0.510 | 0.440 – 0.580 | Lognormal | Estimated from the HR of diabetes incidence among individuals having 2 lifestyle diabetes prevention behaviours. | [11] |
| Risk of prediabetes incidence among individuals having 3 lifestyle behaviours | 0.340 | 0.280 – 0.410 | Lognormal | Estimated from the HR of diabetes incidence among individuals having 3 lifestyle diabetes prevention behaviours. | [11] |
| Risk of prediabetes incidence among individuals having 4 lifestyle behaviours | 0.220 | 0.170 – 0.280 | Lognormal | Estimated from the HR of diabetes incidence among individuals having 4 lifestyle diabetes prevention behaviours. | [11] |
| Undiagnosed T2D prevalence | 0.737 | 0.663 – 0.811 | Beta | Prevalence of the undiagnosed diabetes population among the overall diabetes cases in Indonesia. | [11] |
| Risk of CVD event among undiagnosed T2D | 2.569 | 1.604 - 4.115 | Lognormal | CVD event includes CVD incidence and CVD-related death. Estimated from the hazard ratio of CVD event among dysglecemic population who are hypertensive in the Bogor Cohort Study | [8] |
| Risk of mortality among T2D | 1.890 | 1.740 – 2.040 | Lognormal | Hazard ratio of all-cause mortality among diabetes population. | [9] |
| Risk of mortality among CVD | 2.570 | 2.190 – 3.020 | Lognormal | Hazard ratio of mortality because of CVD among the diabetes population | [9] |
| **Utility value** |  |  |  |  |  |
| Non-diabetes population | 0.940 | 0.930 – 0.940 | Beta | QALYs among participants without prior diabetes diagnosis and with a fasting blood glucose value of less than < 100 mg/dL. | [12] |
| Prediabetes | 0.930 | 0.920 – 0.940 | Beta | QALYs among participants without prior diabetes diagnosis and with a fasting blood glucose value between 100 – 125 mg/dL. | [12] |
| Undiagnosed T2D | 0.890 | 0.850 – 0.920 | Beta | QALYs among participants without prior diabetes diagnosis and with a fasting blood glucose value between > 125 mg/dL. | [12] |
| Diagnosed with T2D, treated at the PHC level | 0.860 | 0.830 – 0.880 | Beta | QALYs among diagnosed T2D who are treated at the PHC and enrolled in *Prolanis* program. | [13] |
| Diagnosed with T2D, treated at the secondary level | 0.800 | 0.760 – 0.830 | Beta | QALYs among outpatient T2D patients treated at both the primary and secondary care levels without any complications. | [14] |
| T2D with CVD complications | 0.790 | 0.720 – 0.820 | Beta | QALYs among outpatient T2D patients treated at both the primary and secondary care levels with macrovascular complications. | [14] |
| **Health State Costs (Int$)** |  |  |  |  |  |
| High risk non-diabetic patients treated at the PHC level. | 59.930 | 41.950 – 77.900 | Gamma | Estimated from the direct medical cost of *Prolanis* program per capita and the yearly capitation cost received by primary health care. | [15, 16] |
| Diagnosed T2D at PHC level | 1,030.780 | 721.540 – 1,340.010 | Gamma | Estimated from the yearly capitation cost received by primary health care, cost of *Prolanis* program per capita, hospitalization, and diabetes and non-diabetes related medication cost | [15–17] |
| Diagnosed with T2D at the secondary level | 1,466.750 | 1,026.730 – 1,906.780 | Gamma | Direct medical costs of T2D patients without complications | [16] |
| T2D having CVD complications | 3,251.300 | 2,275.910 – 4,226.690 | Gamma | Direct medical costs of T2D patients with complications | [16] |
| Training cost for community health workers | 554.900 | 388.430 – 721.370 | Gamma | Median training cost for community health workers per community. | SUNI-SEA qualitative interview with district health officers in 4 cities in Indonesia. |
| Community health workers' financial compensations/incentives | 958.620 | 671.030 – 1,246.210 | Gamma | Median remuneration fee for community health workers per community. | SUNI-SEA qualitative interview with district health officers in 4 cities in Indonesia. |
| Health education material development | 58.560 | 40.990 – 76.130 | Gamma | Median cost to develop health education material per community. | SUNI-SEA qualitative interview with district health officers in 4 cities in Indonesia. |
| Health education application | 5,153.850 | 3,607.700 – 6,700.010 | Gamma | Cost to develop *Posyandu* application | SUNI-SEA cost data |
| Screening kit | 4.120 | 2.880 – 5.360 | Gamma | Cost to provide RCBG screening kit per patient | SUNI-SEA qualitative interview with district health offices in 4 cities in Indonesia. |
| Confirmatory kit | 4.120 | 2.880 – 5.360 | Gamma | Cost to provide a Fasting Blood Glucose test per patient. | Assumed to be the same with the RCBG kit per patient. |

***Notes:** When estimating the annual transition probability from a different time frame (e.g., 6 years), we first converted the probability to a rate using Formula 1. We then adjusted the time to match the time cycle of our Markov model. Lastly, we converted the rate back to a probability using Formula 2.[18] A similar approach was used to estimate the change in transition probability due to the intervention’s effect.

(1) $r=\left( \frac{1}{t} \right)ln(1-p)$

(2) $1-e^{-rt}$

Notes: r = rate; t = time; p = probability

# Scenario Analysis

**Notes:** The current uptake level and adherence to the PHC referral recommendation are 10.7% and 6.0%, respectively.

**Figure F1 ICER values of the one-time screening only strategy, depending on their uptake level and adherence to the referral recommendation**

**Notes:** The current uptake level and adherence to the PHC referral recommendation are 10.7% and 6%, respectively

**Figure F2 ICER values of the one-time screening and health education strategy, depending on their uptake level and adherence to the referral recommendation**

**Notes:** The current uptake level and adherence to the PHC referral recommendation are 10.7% and 6%, respectively.

**Figure F3 ICER values of repeated screening only strategy, depending on their uptake level and adherence to the referral recommendation**

**Notes:** The current uptake level and adherence to the PHC referral recommendation are 10.7% and 6%, respectively.

**Figure F4 ICER values of the repeated screening and health education strategy, depending on their uptake level and adherence to the referral recommendation**


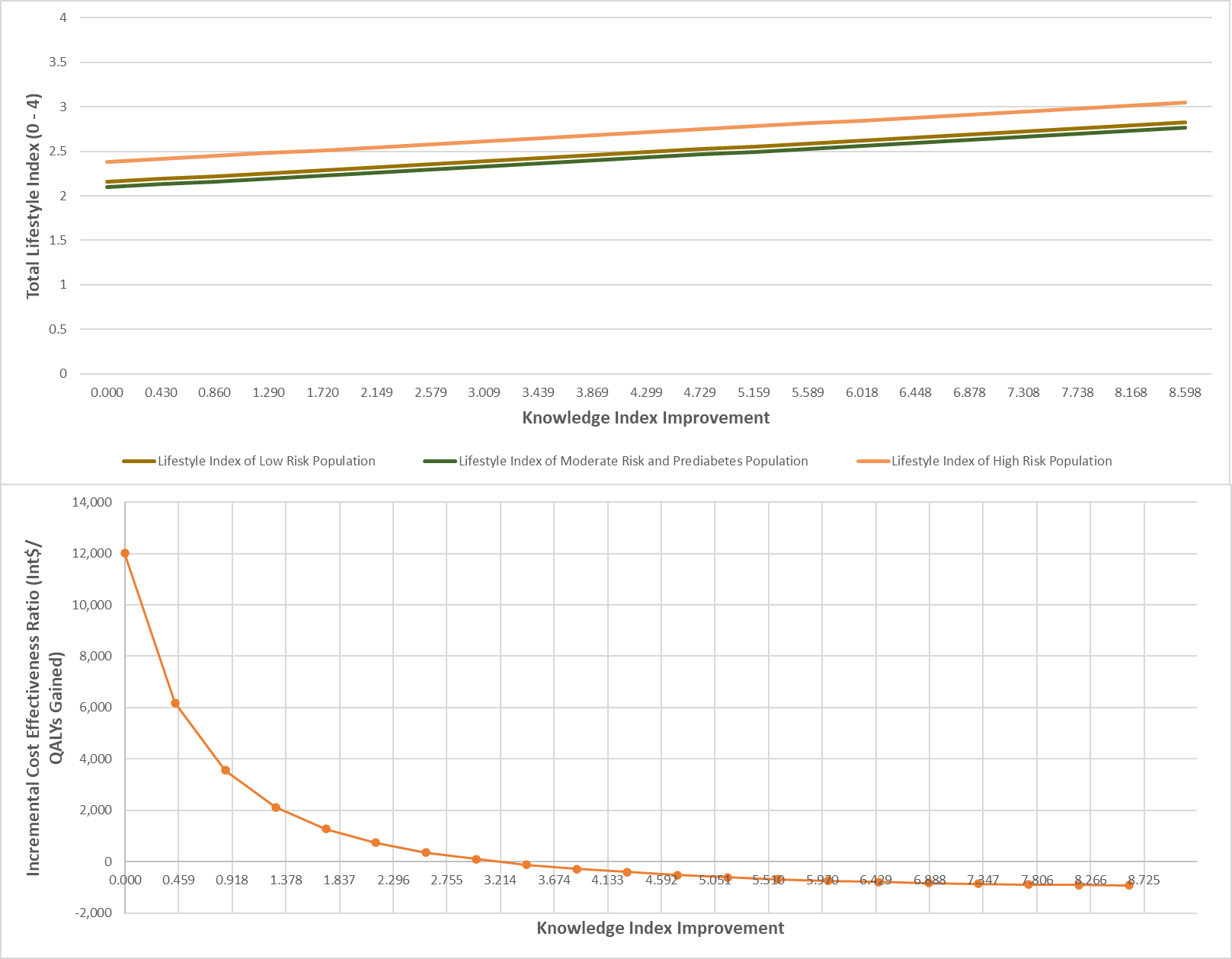


**Notes:** The current knowledge improvement level is 0.860

**Figure F5 Lifestyle index improvement and ICER values for different scenarios of diabetes knowledge improvement**

# One-way sensitivity analysis


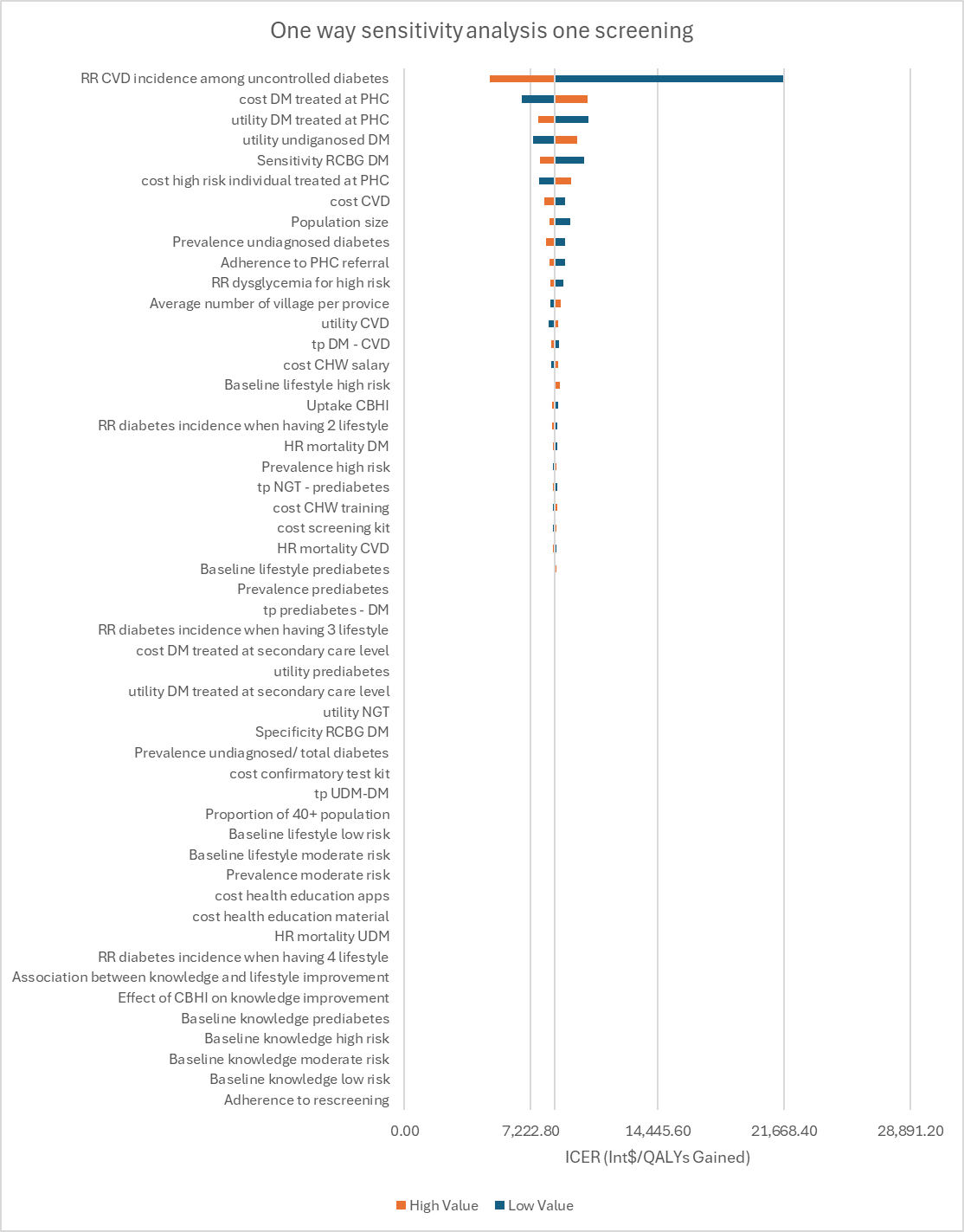


**Figure G1 Contributors on the Uncertainty around the ICER value of One-Time Screening**


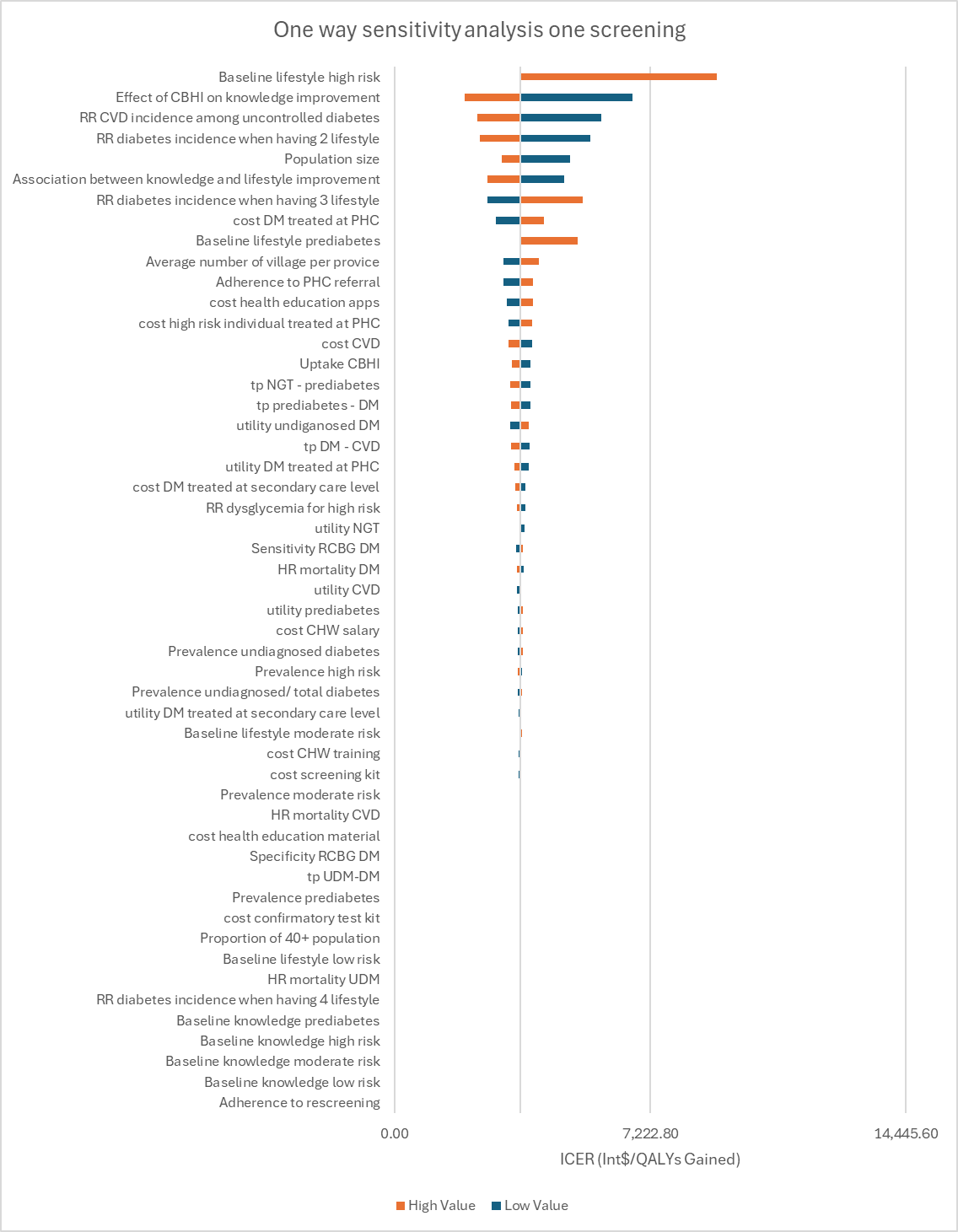


**Figure G2 Contributors to the Uncertainty around the ICER Value of One-time Screening and Health Education**

**
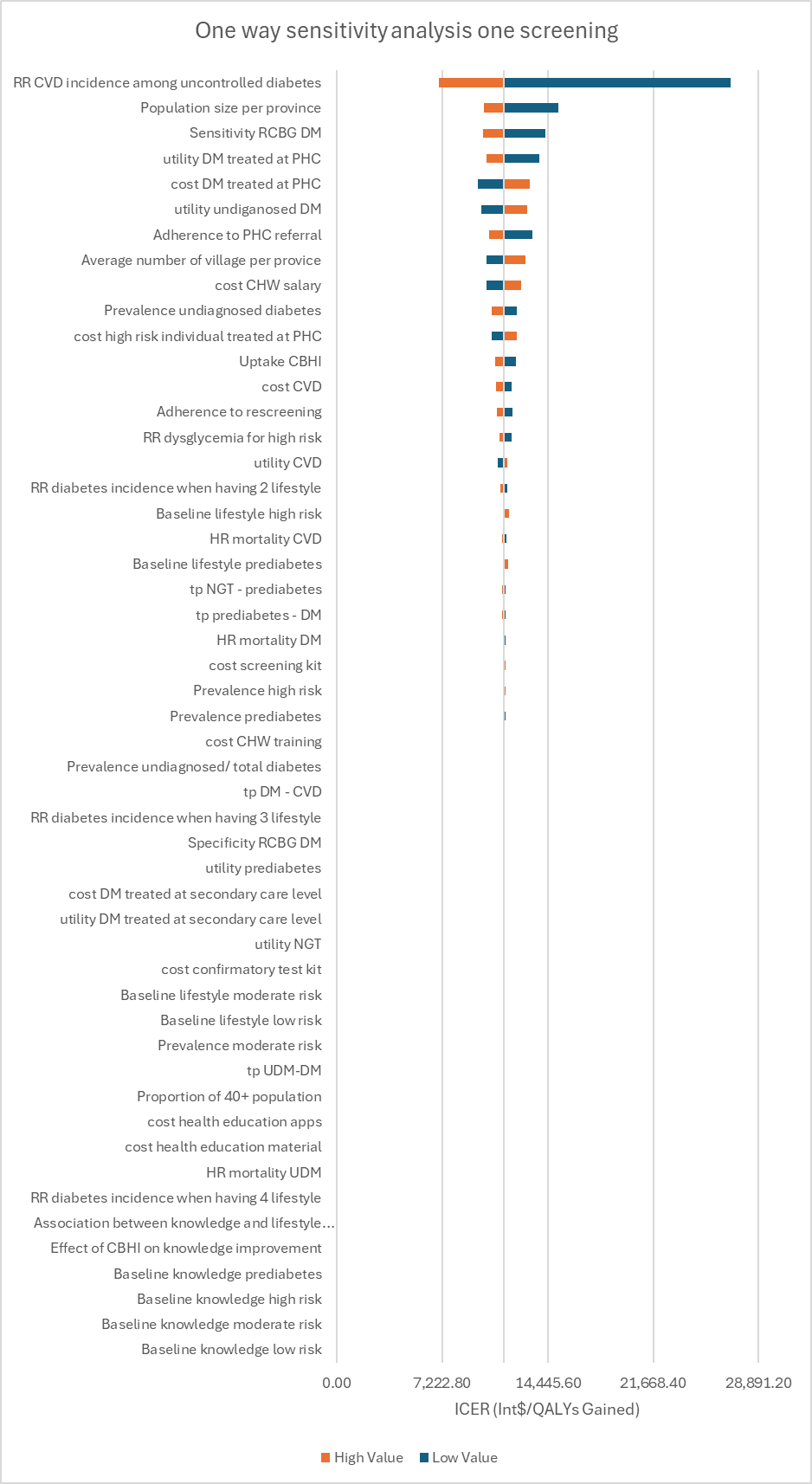
**

**Figure G3 Contributors to the Uncertainty around the ICER Value of Repeated Screening**

**
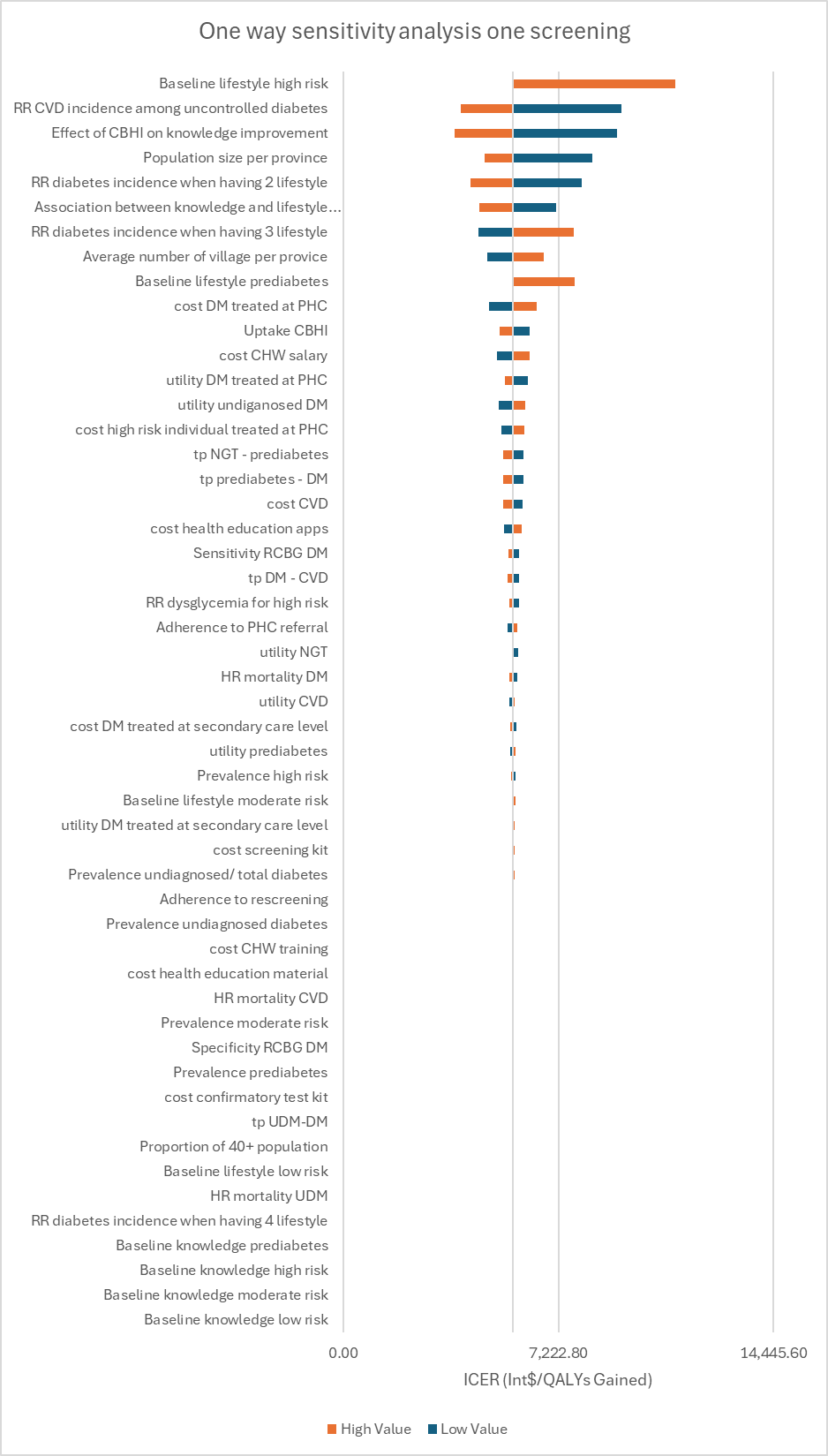
**

**Figure G4 Contributors to the Uncertainty around the ICER Value of Repeated Screening and Health Education**

# References

1. Ministry of Health of Republic of Indonesia. (2023). Keputusan Menteri Kesehatan Republik Indonesia (Regulation of Ministry of Health) No. HK.01.07/MENKES/2015/2023. Ministry of Health of Republic of Indonesia.

2. Directorate of Non-communicable Disease, Ministry of Health of Indonesia. (2017). *Pandu PTM*. Ministry of Health of Republic of Indonesia. Retrieved from https://www.scribd.com/document/385364672/PANDU-PTM-ENGLISH

3. Badan Kebijakan Pembangunan Kesehatan, M. of H. of R. of I. (2023). *Survei Kesehatan Indonesia Dalam Angka (Indonesian Health Survey in Number) 2023*. Jakarta, Indonesia. Retrieved from https://layanandata.kemkes.go.id/katalog-data/ski/ketersediaan-data/ski-2023

4. Somannavar, S., Ganesan, A., Deepa, M., Datta, M., & Mohan, V. (2009). Random Capillary Blood Glucose Cut Points for Diabetes and Pre-Diabetes Derived From Community-Based Opportunistic Screening in India. *Diabetes Care*, *32*(4), 641–643. https://doi.org/10.2337/dc08-0403

5. Anjana, R. M., Shanthi Rani, C. S., Deepa, M., Pradeepa, R., Sudha, V., Divya Nair, H., … Mohan, V. (2015). Incidence of Diabetes and Prediabetes and Predictors of Progression Among Asian Indians: 10-Year Follow-up of the Chennai Urban Rural Epidemiology Study (CURES). *Diabetes Care*, *38*(8), 1441–1448. https://doi.org/10.2337/dc14-2814

6. World Health Organization. (2020). *Lifetables by country: Indonesia*. World Health Organization. Retrieved from https://www.who.int/data/gho/data/themes/topics/topic-details/GHO/healthy-life-expectancy-(hale)

7. Ogurtsova, K., Guariguata, L., Barengo, N. C., Ruiz, P. L.-D., Sacre, J. W., Karuranga, S., … Magliano, D. J. (2022). IDF diabetes Atlas: Global estimates of undiagnosed diabetes in adults for 2021. *Diabetes Research and Clinical Practice*, *183*, 109118. https://doi.org/10.1016/j.diabres.2021.109118

8. Sibarani, M. H. R., Wijaya, I. P., Rizka, A., Soewondo, P., Riyadina, W., Rahajeng, E., … Tahapary, D. L. (2022). Cardiovascular disease prediction model for Indonesian adult population with prediabetes and diabetes mellitus: The Bogor Cohort study of Noncommunicable Diseases Risk Factors. *Diabetes & Metabolic Syndrome: Clinical Research & Reviews*, *16*(1), 102330. https://doi.org/10.1016/j.dsx.2021.102330

9. Yang, J. J., Yu, D., Wen, W., Saito, E., Rahman, S., Shu, X.-O., … Zheng, W. (2019). Association of Diabetes With All-Cause and Cause-Specific Mortality in Asia: A Pooled Analysis of More Than 1 Million Participants. *JAMA Network Open*, *2*(4), e192696. https://doi.org/10.1001/jamanetworkopen.2019.2696

10. Ministry of Health of Republic of Indonesia. (2019). *Indonesian Basic Health Research 2018: Report*. Indonesia: Ministry of Health. Retrieved from https://repository.badankebijakan.kemkes.go.id/id/eprint/3514/1/Laporan%20Riskesdas%202018%20Nasional.pdf

11. Lv, J., Yu, C., Guo, Y., Bian, Z., Yang, L., Chen, Y., … on behalf of the China Kadoorie Biobank Collaborative Group. (2017). Adherence to a healthy lifestyle and the risk of type 2 diabetes in Chinese adults. *International Journal of Epidemiology*, *46*(5), 1410–1420. https://doi.org/10.1093/ije/dyx074

12. Rokhman, M. R., Arifin, B., Broggi, B., Verhaar, A.-F., Zulkarnain, Z., Satibi, S., … van der Schans, J. (2023). Impaired health-related quality of life due to elevated risk of developing diabetes: A cross-sectional study in Indonesia. *PLoS ONE*, *18*(12 December). https://doi.org/10.1371/journal.pone.0295934

13. Prabowo, M. H., Febrinasari, R. P., Pamungkasari, E. P., Mahendradhata, Y., Pulkki-Brännström, A.-M., & Probandari, A. (2023). Health-related Quality of Life of Patients With Diabetes Mellitus Measured With the Bahasa Indonesia Version of EQ-5D in Primary Care Settings in Indonesia. *Journal of Preventive Medicine and Public Health*. https://doi.org/10.3961/jpmph.23.229

14. Arifin, B., Idrus, L. R., van Asselt, A. D. I., Purba, F. D., Perwitasari, D. A., Thobari, J. A., … Postma, M. J. (2019). Health-related quality of life in Indonesian type 2 diabetes mellitus outpatients measured with the Bahasa version of EQ-5D. *Quality of Life Research*. https://doi.org/10.1007/s11136-019-02105-z

15. Khoe, L. C., Wangge, G., Soewondo, P., Tahapary, D. L., & Widyahening, I. S. (2020). The implementation of community-based diabetes and hypertension management care program in Indonesia. *PLOS ONE*, *15*(1), e0227806. https://doi.org/10.1371/journal.pone.0227806

16. Hidayat, B., Ramadani, R. V., Rudijanto, A., Soewondo, P., Suastika, K., & Siu Ng, J. Y. (2022). Direct Medical Cost of Type 2 Diabetes Mellitus and Its Associated Complications in Indonesia. *Value in Health Regional Issues*, *28*, 82–89. https://doi.org/10.1016/j.vhri.2021.04.006

17. Ministry of Health of Republic of Indonesia. (2023). Standard Health Service Tariffs in the Implementation of Health Insurance Programs. Ministry of Health of Republic of Indonesia. Retrieved from https://farmalkes.kemkes.go.id/unduh/permenkes-3-2023-standar-tarif-pelayanan-kesehatan-dalam-penyelenggaraan-program-jaminan-kesehatan/

18. Gray, A., Clarke, P. M., Wolstenholme, J. L., & Wordsworth, S. (2011). *Applied methods of cost-effectiveness analysis in health care*. Oxford: Oxford University Press.
